# Individual-Based Ant-Plant Networks: Diurnal-Nocturnal Structure and Species-Area Relationship

**DOI:** 10.1371/journal.pone.0099838

**Published:** 2014-06-11

**Authors:** Wesley Dáttilo, Roberth Fagundes, Carlos A. Q. Gurka, Mara S. A. Silva, Marisa C. L. Vieira, Thiago J. Izzo, Cecília Díaz-Castelazo, Kleber Del-Claro, Victor Rico-Gray

**Affiliations:** 1 Instituto de Neuroetología, Universidad Veracruzana, Xalapa, Veracruz, Mexico; 2 Laboratório de Ecologia Comportamental e Interações, Instituto de Biologia, Universidade Federal de Uberlândia, Uberlândia, Brazil; 3 Universidade Federal de Ouro Preto, Ouro Preto, Brazil; 4 Universidade Federal de Mato Grosso, Cuiabá, Brazil; 5 Red de Interacciones Multitróficas, Instituto de Ecología A.C. Xalapa, Veracruz, México; Arizona State University, United States of America

## Abstract

Despite the importance and increasing knowledge of ecological networks, sampling effort and intrapopulation variation has been widely overlooked. Using continuous daily sampling of ants visiting three plant species in the Brazilian Neotropical savanna, we evaluated for the first time the topological structure over 24 h and species-area relationships (based on the number of extrafloral nectaries available) in individual-based ant-plant networks. We observed that diurnal and nocturnal ant-plant networks exhibited the same pattern of interactions: a nested and non-modular pattern and an average level of network specialization. Despite the high similarity in the ants’ composition between the two collection periods, ant species found in the central core of highly interacting species totally changed between diurnal and nocturnal sampling for all plant species. In other words, this “night-turnover” suggests that the ecological dynamics of these ant-plant interactions can be temporally partitioned (day and night) at a small spatial scale. Thus, it is possible that in some cases processes shaping mutualistic networks formed by protective ants and plants may be underestimated by diurnal sampling alone. Moreover, we did not observe any effect of the number of extrafloral nectaries on ant richness and their foraging on such plants in any of the studied ant-plant networks. We hypothesize that competitively superior ants could monopolize individual plants and allow the coexistence of only a few other ant species, however, other alternative hypotheses are also discussed. Thus, sampling period and species-area relationship produces basic information that increases our confidence in how individual-based ant-plant networks are structured, and the need to consider nocturnal records in ant-plant network sampling design so as to decrease inappropriate inferences.

## Introduction

Ants and plants can interact in different ways, from facultative to highly specialized relationships [1]. For instance, extrafloral nectar-mediated ant–plant mutualisms are among the most remarkable ecological interactions in terrestrial ecosystems [2]. In this type of ant-plant interaction, plants with extrafloral nectaries (EFN-bearing plants) produce a liquid rich in carbohydrates and amino acids, which attracts different ant species [3]. In exchange for food, some ants can protect the host plant against potential herbivores [1]. At the community level, different ant and plant species can interact with each other generating complex ecological networks; where plant and ant species are depicted as nodes and their interactions are depicted by links [4], [5], [6].

Focused on the structure of ant-plant ecological networks, some studies have found some non-random patterns of interactions around the world as, for example, the nested pattern [4], [7], [8]. This pattern indicates that within an ant-plant network there is a core of generalist species (those with the most interactions), which interact among themselves, and specialists species (those with fewer interactions) also interacting with the generalist species in cohesive subgroups [9]. Another characteristic of these facultative ant-plant networks is that they do not exhibit a modular pattern of interactions, since there are no groups of ants specialized in feeding on a given group of plants [10], [11]; as previously demonstrated in symbiotic networks involving ants and myrmecophytes [12]. However, despite the importance and increasing knowledge of ant-plant networks at the community level, no study has evaluated how intrapopulation variation in plants can contribute to the organization of the ant species associated to individual EFN-bearing plants. As a single EFN-bearing plant can be associated to several ant species in a predictable way, we can also use a network approach to evaluate the structure of such individual-based ant-plant networks.

We already know that within an EFN-bearing plant population the richness of associated ants differs among plants [13], [14], [15] One of the main factors explaining this intrapopulation variation is based on the difference in the reward offered (quantity and quality of nectar), where individuals with better rewards would be most visited by ants [16], [17]. On the other hand, nectar quantity and quality can vary along the day, and on the same plant [18], [19], [20], which means that an individual plant may be not considered to be a good resource throughout the day. Therefore, this variation in nectar production can influence the daily rate of ant foraging [20], and colony growth [21]. In addition, foraging of some ant species, including those that forage on EFN-bearing plants, can be strictly diurnal, nocturnal, or both [22], [23], Therefore, there are good reasons to expect temporal turnover of species composition of ant assemblages and intrapopulation variation in ant-plant networks.

An individual EFN-bearing plant can be viewed as an “island of resources” for ants and compared with the classical model of species-area relationship proposed by MacArthur and Wilson [24]. In ant-plant interactions, it is possible to expect an increase of ant richness throughout plant ontogeny, mainly due to higher availability of food resources for ants (*e.g.,* number of extrafloral nectaries-EFNs) [25], [26], [27]. In fact, bigger and older plants can increase their nutrient-acquiring and photosynthetic capacity and invest in biotic defense via extrafloral nectaries [28]. The increase in the richness of ant occurs mainly because bigger plant individuals with higher number of EFNs would allow for a greater spatial segregation in the use of their food resources, which increases ant coexistence [27]. Thus, the difference in ants’ presence on plants from the same population is linked not only by available food resources, but also by the foraging time per ant species and species-area relationships. This intrapopulation variation can directly affect the fitness of each individual plant [29] since it is expected that, in general, the most visited individuals can be better protected against herbivores [30].

Here, using both diurnal and nocturnal sampling, we evaluated the network structure and species-area relationships in individual-based ant-plant networks. Specifically, due to nectar production features and ant foraging shifts between day and night, we hypothesized that there would be changes in the composition and in the specific positions of ants within the network when diurnal and nocturnal networks were compared. Moreover, based on species-area relationship, we also postulated that plant individuals with higher number of EFNs had a greater richness of associated ants (*i.e.*, greater degree within a nested network). In order to test our hypotheses, we conducted diurnal and nocturnal sampling of ants interacting with three plant species in different regions in the Brazilian Neotropical savanna.

## Materials and Methods

### Ethics Statement

All necessary permits were obtained for the described field studies. All research was conducted with the approval of the Brazilian Chico Mendes Institute for Biodiversity Conservation (ICMBIO/SISBIO, Permit Number 12427/2007) and the Minas Gerais Forest Institute (IF/MG, Permit Number 064/11).

### Study Area and Species Studied

**Figure 1:**
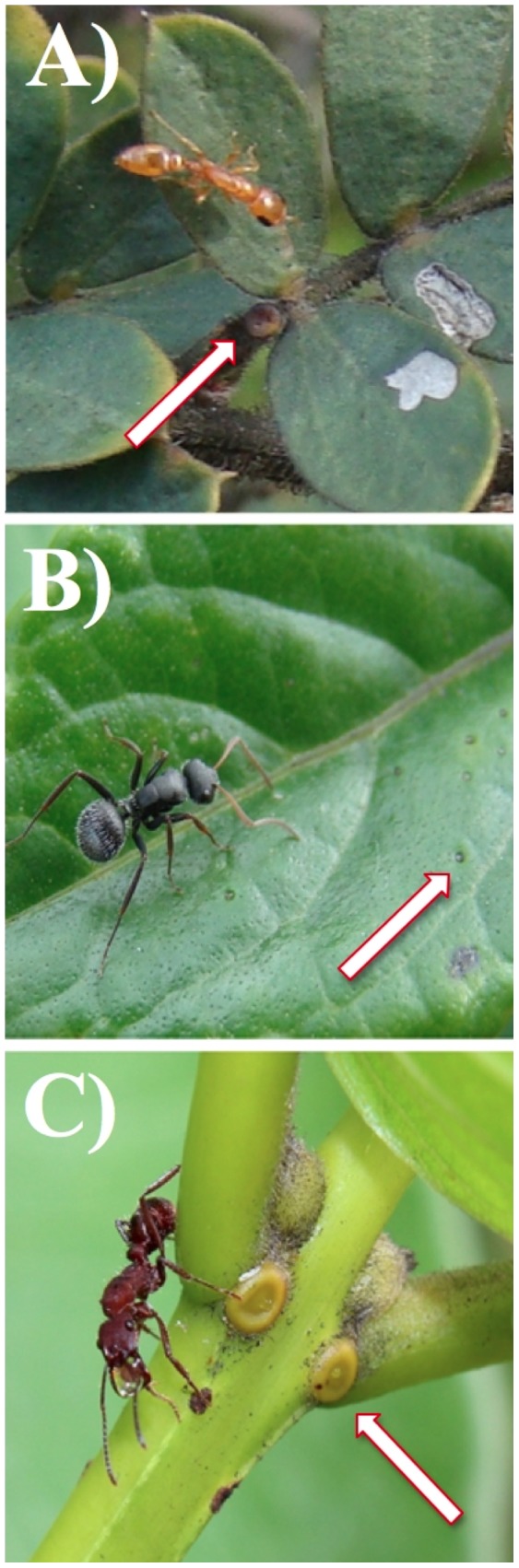
Ants foraging on plant species bearing extrafloral nectaries studied in the Brazilian Neotropical savanna: A) *Chamaecrista mucronata* (Leguminosae–Caesalpinioideae), B) *Stachytarpheta glabra* (Verbenaceae), and C) *Qualea grandiflora* (Vochysiaceae). Ant foragers shown are: A) *Pseudomyrmex pallidus* (Pseudomyrmecinae), B) *Camponotus crassus* (Formicinae), and C) *Ectatomma tuberculatum* (Ectatomminae). Arrows indicate the position of the extrafloral nectaries.

We sampled individual-based ant-plant networks in three different localities of the Brazilian Neotropical savanna (Cerrado biome). In each field site we observed and annotated the interactions between individuals of one EFN-bearing plant species and their associated ants. As the plants’ composition changes geographically, is not logistically possible to access the same species in all sites, so we choose the three most common EFN-bearing plant species in each site: *Chamaecrista mucronata* (Leguminosae-Caesalpinioideae), *Stachytarpheta glabra* (Verbenaceae), and *Qualea grandiflora* (Vochysiaceae) (Figure 1).

We quantified the interactions between ants and *S. glabra* in July of 2011 and *C. mucronata* in September 2011 at Parque Estadual do Itacolomi (20°26’S and 43°30’W, for *S. glabra* and 20°24′S and 43°30′O, for *C. mucronata*), located in Ouro Preto, in the center of State of Minas Gerais, Brazil. The regional climate, according to Köppen classification is tropical savanna (Cwa) with a mean annual temperature of 21°C and 1700 mm of precipitation. The park covers an area of ca.7000 ha with different savanna physiognomies (44%) [31]. *S. glabra* is the most common species in the field site, with mean height of 1.5 to 2 m with very small extrafloral nectaries spreading on the upper leaf surface (Antonini et al. 2005). *C. mucronata* is one of the most common species of *campos rupestres* (rupestrian fields). *C. mucronata* is a highly branched shrub with average height of 0.8–1 m, presenting compound leaves with 6–8 leaflets, and one nectary per leaf [32]. Rupestrian fields are characterized by tortuous trees and shrubs immersed in open fields of grasses and rocky outcrop [32].

We sampled the interactions between ants and individuals of *Q. grandiflora* in November 2013 at Estação Ecológica Serra das Araras (EESA) (15°38′S and 57°12′W), located in the municipality of Porto Estrela, state of Mato Grosso, Brazil. The regional climate, according to Köppen classification is tropical savanna (Cwa) with a mean annual temperature of 28°C and 1400 mm of precipitation. The reserve area covers 28,700 ha of continuous forest with different savanna physiognomies [33]. *Q. grandiflora* is a typical and abundant woody tree of the Brazilian Neotropical savanna which reaches up to 15 m tall, bearing paired oval and elevated extrafloral nectaries on the stem next to the insertion of the leaves, and on the bud pedicels [34].

### Data Collection

We assessed the interactions between ants and plants along one transect of 300 m × 10 m for *C. mucronata* and *S. glabra* and 500 m × 10 m for *Q. grandiflora*. Within each transect in each of three different sites, we checked all individuals of the three species considered and recorded all ants collecting nectar from their EFNs. We used a ladder to sample branches of a few larger individuals. We marked all sampled individuals during the day to be reviewed at night. Ant-plant interactions were sampled in two time intervals: Diurnal sampling: 08∶00–12∶00, and Nocturnal sampling: 20∶00–23∶00. However, the number of plants sampled could vary among samplings, since not all plants sampled in the day had ants at night, and *vice versa*. Moreover, no plants with hemipterans or any other visible liquid-resource were included in our sampling. This restriction was made in order to avoid ant aggregation caused by attraction to another kind of food resource. We also recorded the maximum height of each individual (ground to canopy) at the time of each collection. In this study, we used the number of EFNs rather than plant height to evaluate species-area relationships, mainly because the number of EFNs would make a more robust indicator of resource availability for ants. For this, we estimated the number of EFNs per plant based on the method proposed by Blüthgen et al. [25], which consists in counting the number of EFNs of three random branches, extracting the mean value, and then multiplying by the number of total branches in the plant. This procedure was done for each individual of *C. mucronata* and *Q. grandiflora*. In cases where plants had a variable number of EFNs per leaf (*i.e., S. glabra*), we calculated the mean number of EFNs using ten random leaves per branch (three branches), and then we multiplied by the number of total branches in the plant.

Ant specimens were identified at the lowest possible taxonomic level with the assistance of identification keys available in the literature and by morphological comparisons of ant species deposited in our reference collections. Ant vouchers were deposited in the: Coleção Entomológica do Departamento de Biodiversidade Evolução e Meio Ambiente da Universidade Federal de Ouro Preto for ants associated to *C. mucronata* and *S. glabra,* and Coleção Entomológica of Universidade Federal de Mato Grosso (CEMT) for ants associated to *Q. grandiflora*.

### Network Analysis and Statistics

We used each plant species and associated ants as independent ant-plant networks. Each individual-based ant-plant network was built by an adjacency matrix ***A***, where *a_ij_* = number of interaction from an individual plant *j* by the ant species *i*, and zero otherwise [9]. For each plant species (*Chamaecrista mucronata*, *Stachytarpheta glabra*, and *Qualea grandiflora*), we build interaction ant-plant networks according to the sampling period (diurnal, nocturnal, or both together), totaling nine networks.

We first evaluated the specialization for each of the nine ant-plant networks using an index extremely robust to changes in sampling intensity and the number of interacting species called: *H_2_*’ [35], [36]. In this index, extreme generalization of an ecological network is *H_2_*’ = 0 and extreme specialization is *H_2_*’ = 1. Then we performed a second approach that involves the search for non-random patterns of interactions commonly found in ant-plant networks studied at the community level. Specifically, we evaluated if selective ant species would visit only a subset of plant individuals visited by the generalist ant species (*i.e.*, nested pattern of ant-plant interactions). For this, we estimated nestedness using the NODF-metric [37] in the ANINHADO program, a program developed by Guimarães and Guimarães [38] to perform rapid and automatic calculation of nestedness in bipartite matrices. The values of this metric range from 0 (non-nested) to 100 (perfectly nested). NODF-values are less prone to Type I statistical error when compared to other nestedness indices [37]. Moreover, we tested whether within each ant-plant network there were groups of ant species strongly associated with a particular set of individual plants, as expected in a modular network. For this we used the modularity index (*M*) based on Simulated Annealing (*SA*) (range 0–1) [39], [40] using the software MODULAR [41]. This index ranges from 0, no subgroups, to 1, totally separated subgroups [42]. We generated random matrices to test the significance of modularity and nestedness according to the Null Model II (CE) [9] using functions within the software MODULAR and ANINHADO (n = 1000 randomizations for each network). In this null model, the probability of occurrence of an interaction is proportional to the number of interactions of both ant species and plant individuals [9]. We used these network descriptors and null model because they provide a way to characterize the organization of these networks and allows direct comparison with previous works on ant-plant networks. We used the recent formula proposed by Dáttilo et al. [6] to describe ant species as peripheral (selective species, those with fewer interactions) or generalist core (generalist species, those with the most interactions) components of the networks: *Gc* =  (*k_i_* − *k_mean_*)/*σ_k_*, where *k_i_* = mean number of links for a given ant species, *k_mean_* = mean number of links for all ant species in the network, and *σ_k_* = standard deviation of the number of links for ant species. *Gc* >1 are ant species of the generalist core, and *Gc* <1 are peripheral ant species. This categorization enabled us to evaluate the temporal turnover in the specific positions of ants within each network (*e.g.*, shifting from peripheral to generalist core between diurnal and nocturnal networks). Moreover, we computed Jaccard’s similarity index (*JSI*) for each network in order to explore the turnover in ant composition between diurnal and nocturnal networks (see [43]). Jaccard’s similarity index between *D* (diurnal) and *N* (nocturnal) network was computed as follows: JSI_ (*DN*)_ = *A*/(*A*+*B*+*C*), where *A* is the number of ant species shared between the two sampling time periods, *B* is the number of ant species present only in the first period, and *C* is the number of ant species present only in the second period.

Finally, to test our hypothesis that the number of EFNs could increase the richness of associated ants (*i.e.*, greater degree within a nested network), for each individual-based ant-plant network studied, we used Simple Linear Regressions (SLR) with the number of EFNs as predictor variable and ant richness as a dependent variable. However, before performing SLR analysis, we used a one-sample Student *t*-test to evaluate the statistical variation of our predictor variable (number of EFNs) around their own mean for all plant species (all *p*-values <0.05). This analysis is especially important to assess whether the number of EFNs varied enough in our individual plants to be able to influence the richness of associated ants. We did SLR analysis and Student’s *t*-tests using the *vegan* package [44] in the R–software version 2.1.3.1 [45].

## Results

We recorded 32 ant species representing 13 genera and six subfamilies (Appendix S1) interacting with the three studied plant species.

**Figure 2:**
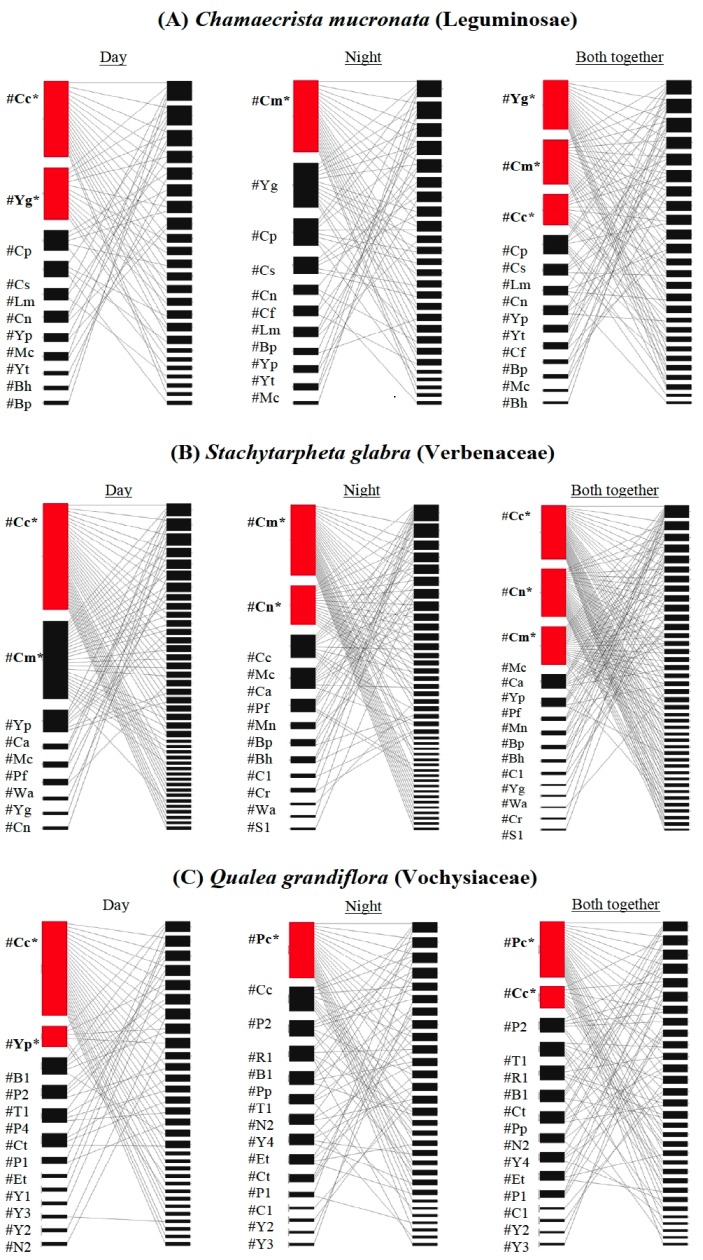
Individual ant–plant networks sampled in the Brazilian Neotropical Savanna involving for three plant species: (A) *Chamaecrista mucronata* (Leguminosae–Caesalpinioideae), (B) *Stachytarpheta glabra* (Verbenaceae), and (C) *Qualea grandiflora* (Vochysiaceae). For each plant species we built ant–plant networks using diurnal, nocturnal, and both together sampling. Within each network, node represents one ant species (left) or plant individual (right), and lines represent ant–plant interactions. The nodes are arranged according their position in the nestedness ranking. Rectangle height is proportional to the number of interactions of each species. Asterisks (*) and red rectangles denote ant species that were present in the generalist core. Species codes are in Appendix S1.

In the diurnal individual-based network of *Chamaechrista mucronata*, we recorded 11 ant species (two exclusive) foraging on nectaries of 24 individuals, and 11 ant species (two exclusive) on 26 individuals in the nocturnal network (Figure 2A) (n = 26 individuals were sampled in both periods). The total ant richness (the two periods of sampling) was 13 species. Mean ant richness per plant individual was similar in the diurnal (Mean ± SD: 2.12±1.03) and nocturnal (2.03±1.08) networks. However, when the two sampling periods were combined, there was an increase in the mean ant richness per individual (3.14±1.61). In addition, despite the high similarity in ant composition between diurnal and nocturnal networks (Jaccard similarity index = 0.866), the ant assemblage present in the generalist core (*i.e.*, those with the most links) totally changed between the two sampling periods. The ant species *Camponotus crassus* (Formicinae) and *Pseudomyrmex gracillis* (Pseudomyrmecinae) were part of the generalist core in the diurnal networks (18.1% of the total ant species sampled), while only *Camponotus melanoticus* (Formicinae) was part of the generalist core in the nocturnal networks (9.09% of the total ant species sampled). Whereas, three ant species were part of the generalist core including both diurnal and nocturnal sampling: *C. crassus*, *C. melanoticus*, and *P. gracillis* (23% of the total ant species sampled). In addition, we observed an average level of network specialization in both diurnal and nocturnal networks (Diurnal: *H_2_’* = 0.519. Nocturnal: *H_2_’* = 0.499). However, the level of network specialization decreased when we included both diurnal and nocturnal data (*H_2_’* = 0.409) (Table 1).

**Table 1:** Networks descriptors for individual–based ant–plant networks involving three plant species: *Chamaecrista mucronata* (Leguminosae–Caesalpinioideae), *Stachytarpheta glabra* (Verbenaceae), and *Qualea grandiflora* (Vochysiaceae).

	Day	Night	Whole–Day
***Chamaecrista mucronata***			
Plant individuals sampled	24	26	26
Plant individuals exclusively sampled	0	2	–
Mean height	2.41±0.78	2.32±0.82	2.32±0.82
Number of extrafloral nectaries	499.76±447.66	531.03±489.93	531.03±489.93
Ant richness	11	11	13
Exclusive ant species	2	2	–
Mean of ant richness per individual	2.12±1.03	2.03±1.08	3.14±1.61
Ant species in the generalist core	2	1	3
Network specialization	0.519	0.499	0.409
Nestedness^a^	46.93	46.29	48.42
Modularity^b^	0.388	0.359	0.293
***Stachytarpheta glabra***			
Plant individuals sampled	41	41	41
Plant individuals exclusively sampled	0	0	–
Mean height	2.71±1.73	2.82±1.71	2.82±1.71
Number of extrafloral nectaries	52137.95±82818.58	52137.95±82818.58	52137.95±82818.58
Ant richness	9	13	15
Exclusive ant species	2	6	–
Mean of ant richness per individual	1.85±0.89	2.21±1.51	3.02±1.35
Ant species in the generalist core	2	2	3
Network specialization	0.494	0.518	0.401
Nestedness^a^	58.62	43.29	54.75
Modularity^b^	0.319	0.394	0.301
***Qualea grandiflora***			
Plant individuals sampled	31	30	31
Plant individuals exclusively sampled	1	0	–
Mean height	1.64±0.66	1.60±0.38	1.64±0.66
Number of extrafloral nectaries	375.89±152.83	381.87±151.75	375.89±152.83
Ant richness	13	15	15
Exclusive ant species	0	2	–
Mean of ant richness per individual	1.87±0.84	2.22±1.11	2.54±0.99
Ant species in the generalist core	2	1	2
Network specialization	0.509	0.499	0.424
Nestedness^a^	58.62	27.84	30.16
Modularity^b^	0.434	0.421	0.391

Values indicate Mean ± SD. Sampling of ant–plant interactions were performed in the Brazilian Neotropical Savanna (see text for more information).

a All ant–plant networks were significantly nested (P<0.05).

b No ant–plant network showed a modular pattern of interaction (P>0.05).

In the diurnal individual-based network of *Stachytarpheta glabra*, we recorded nine ant species (two exclusive) foraging on nectaries of 41 individuals, and 13 ant species (six exclusive) on 41 individuals in the nocturnal network (Figure 2B) (n = 41 individuals were sampled in both periods). The total ant richness (two sampling periods) was 15 species. Mean ant richness per plant individual was similar in the diurnal (Mean ± SD: 1.85±0.89) and nocturnal (2.21±1.51) networks. However, when the two sampling periods were combined, there was an increase in the mean ant richness per individual (3.02±1.35). In addition, despite the high similarity in ant composition between diurnal and nocturnal networks (Jaccard similarity index = 0.722), the ant assemblage present in the generalist core totally changed between the two sampling periods. The ant species *C. crassus* and *Pseudomyrmex pallidus* (Pseudomyrmecinae) were part of the generalist core in the diurnal networks (22.2% of the total ant species sampled), while *C. melanoticus* and *Camponotus novogranadensis* (Formicinae) were part of the generalist core in the nocturnal networks (15.3% of the total ant species sampled). Whereas, three ant species were part of the generalist core including both diurnal and nocturnal sampling: *C. crassus*, *C. melanoticus*, and *C. novogranadensis* (23% of the total ant species sampled). In addition, we observed an average level of network specialization in both diurnal and nocturnal networks (Diurnal: *H_2_’* = 0.494. Nocturnal: *H_2_’* = 0.518). However, the level of network specialization decreased when we included both diurnal and nocturnal data (*H_2_’* = 0.401) (Table 1).

In the diurnal individual-based network of *Qualea grandiflora*, we recorded 13 ant species (no exclusive) foraging on nectaries of 31 individuals, and 15 ant species (two exclusive) on 30 individuals in the nocturnal network (Figure 2C) (n = 31 individuals were sampled in both periods). The total ant richness (two sampling periods) was 15 species. Mean ant richness per plant individual was similar in the diurnal (Mean ± SD: 1.87±0.84) and nocturnal (2.22±1.1) networks. However, when the two sampling periods were combined, there was an increase in the mean ant richness per individual (2.54±0.99). In addition, despite the high similarity in ant composition between diurnal and nocturnal networks (Jaccard similarity index = 0.882), the ant assemblage present in the generalist core totally changed between the two sampling periods. The ant species *C. crassus* and *P. pallidus* were part of the generalist core in the diurnal networks (15.3% of the total ant species sampled), while only the giant tropical ant *Paraponera clavata* (Ponerinae) was part of the generalist core in the nocturnal networks (6.6% of the total ant species sampled). Whereas, two ant species were part of the generalist core including both diurnal and nocturnal sampling: *C. crassus* and *P. clavata* (13.3% of the total ant species sampled). In addition, we observed an average level of network specialization in both diurnal and nocturnal networks (Diurnal: *H_2_’* = 0.509. Nocturnal: *H_2_’* = 0.499). However, the level of network specialization decreased when we included both diurnal and nocturnal data (*H_2_’* = 0.424) (Table 1).

Evaluating non-random patterns of ant-plant interaction within each network, we observed that all individual-based networks for the three plant species studied, exhibited a significantly nested network topology (NODF values ranging from 27.84 to 58.62. all *p*-values <0.05), suggesting that the interactions recorded for plant individuals scarcely visited by ants are a cohesive subset of the interactions found on the most visited individuals. In addition, no network was significantly modular when compared with the neutral patterns of ant-plant interactions (null models) (*M* values ranging from 0.293 to 0.421. all *p*-values >0.05) (Table 1), and therefore, there is no group of ant species that feed specifically on a particular group of plant individuals. Finally, when we tested the hypothesis of species-area relationship, we observed that increasing the number of EFNs does not cause an increase in ant richness (*i.e.,* number of links) foraging on such individuals in any of the studied networks (Simple Linear Regression: all *p*-values >0.05).

## Discussion

Using an intrapopulation approach and three plant species, we evaluated the structure of individual-based ant-plant networks in both diurnal and nocturnal sampling in the Brazilian Neotropical savanna. Our results show that diurnal ant-plant networks exhibited the same pattern of interactions when compared with nocturnal networks. However, despite the high similarity in ant composition between the two sampling periods, ant composition in the generalist core totally changes between diurnal and nocturnal sampling for all plant species studied. Whereas, we did not observe any effect of the number of EFNs on ant richness foraging on plants in any of the studied ant-plant network.

Studies dealing with ecological interaction networks at the community level are steadily growing in the literature [46], [47], [48], although only a few address intrapopulation variations within ecological networks [49], [50], [51]. Furthermore, there is an on-going discussion on the standardization of sampling efforts [52] and, in some cases, additional nocturnal sampling [53]. Here we show that individual-based ant-plant networks exhibited the same patterns of interactions found in networks involving ants and EFN-bearing plants studied at the ecological community level: a nested and non-modular pattern of ant-plant interactions and an average level of network specialization [7], [8], [36], [43], [54]. Interestingly, these non-random patterns were stable in all our intrapopulation ant-plant networks regardless of the sampling period (diurnal, nocturnal, or both periods together).

At the community level, we know that different factors can structure both nested and non-modular patterns into ant-plant networks, for example: temperature, precipitation, soil pH, canopy openness, nectar features and phenology, abundance, body size, and dominance hierarchy among ants [8], [11], [55], [56], [57], [58], [59], [60], [61]. So, why do individual-based ant-plant networks exhibit similar patterns of ant-plant interactions when compared to ant-plant networks evaluated at the community level? We observed that species of the genus *Camponotus* were the most extreme generalist ant species in our networks, followed by *Pseudomyrmex* and *Paraponera*. Except for *Paraponera*, all other genera are particularly abundant in the Brazilian Neotropical savanna [62]. Moreover, *Camponotus*, *Pseudomyrmex* and *Paraponera* are strongly associated with EFN-bearing plants [63], [64]. Such tree-dwelling genera are highly aggressive, occupy large territories, have mutually exclusive patterns of distribution within an environment, and use diversified strategies to monopolize this highly nutritive resource [63], [65], [66], [67]. Therefore, based on our knowledge of natural history in the field and literature (see information above), we hypothesize that dominance hierarchy could really be the main mechanism that structures the nested pattern in our ant-plant networks [60]. Competitive dominance can occur by: 1) numerical dominance, as observed for *Camponotus* [1]; or 2) aggressive displacement of competitors by solitary foragers, as in *P. clavata* (pers. obs.). In both cases, dominant ant species would visit most plants in an environment (*i.e.*, generalist core components) while submissive and subordinate ant species co-occur with dominant species only in a few plants [60], [68], [69], [70] Thus, this pattern of ant-plant interactions would generate the cohesive subgroups expected in nested intrapopulation networks. Additionally, the lack of a modular pattern in our ant-plant networks may be explained because the resource offered by EFN-bearing plants is seasonal over space-time, ant species do not exhibit “fidelity” of foraging on the same group of individual plants [7], [23], [67]. Therefore, when a plant does not secrete nectar, the ants can use other resources available on the foliage [71], and therefore interactions tend to be more generalized [67].

When we included both diurnal and nocturnal sampling together, we observed a decrease in network specialization for all plant species. This was possibly due to the increase in the mean richness of associated ants per individual plant caused by the entrance of new highly generalized ant species. Using both diurnal and nocturnal sampling (greater sampling effort), we should expect an increase in the number of species and interactions [52] and, consequently, an increase in the probability to find unique interactions and ant species foraging in only particular period (*i.e.,* ‘forbidden links’) [53], [72]. The record of these forbidden links can exert a strong effect on the structure of an ecological network [73], [74], [75], mainly because it reduces potential sampling bias, as for example, the record of only spatially abundant species. We know that ant foraging can be strictly diurnal, nocturnal, or both [22], including ants that feed on EFN-bearing plants [23], [55]. The temporal partitioning in the daily rhythm of ant foraging on extrafloral nectaries of all three plant species studied by us could directly reduce competitive interactions among sympatric ant species [63], [76], and promote coexistence of greater number of ant species foraging on the same plant individual. Therefore, when recording ant-plant interactions in different sampling periods, we can increase our understanding of the true role of ant species within an ecosystem.

In our study, some ant species changed their role in network structure, shifting from peripheral to generalist core between diurnal and nocturnal sampling. In other words, this “night-shift” indicates that the ecological dynamics of these ant-plant interactions can be temporally partitioned (day and night) at a small spatial scale. For example, in sites with high temperature and low humidity during the day (*e.g.,* Brazilian Neotropical savanna and deserts) due to ecophysiological limitations there is a strong difference in the composition and abundance of ants and herbivores over a 24 h period [77], [78]. We believe this temporal turnover of ant composition in the same individual plant could offer a better protection against a wider range of herbivores, since the protection ability of the host plant can vary among associated ant species [1]. In other words, it is possible that in some cases, processes shaping mutualistic networks formed by protective ants and plants may be underestimated by diurnal sampling alone, possibly because most herbivore activity is also during the night, when weather conditions are milder, that is, temperature falls to 20–22°C and humidity increases to 0–60%.

Despite a significant variation in the number of EFNs, our hypothesis that plant individuals with higher number of EFNs have greater richness of associated ants was not supported. At least two alternative and non-mutually exclusive hypotheses could explain this pattern: 1) due to extrafloral nectar being a highly nutritive and predictable food resource, competitively superior ants could dominate this resource and allow the coexistence of only a few other ant species [68], [69], and 2) due to the limited number of workers in an ant colony, it is possible that the dominant species can only monopolize low trees and with low or hardly any canopy connectivity (*i.e.*, resource access) [79]. In the first case, ant richness would not increase during plant ontogeny (number of EFNs), because competitively superior and territorial ground-nesting ant species could limit the access to the EFN-bearing plants by submissive ant species [1], [30]. This pattern can be stronger in environments where plants are relatively low and with few “active” nectaries. Therefore, most of EFN-bearing plants in these environments could support only a few ant species. In the second case, plants are relatively low in the Brazilian Neotropical savanna, for instance, in our study the largest tree was 7.43 m tall. However, in environments where tree height may reach up to 50 m (*e.g.*, some tropical rainforests) [80], it is expected that different ant species can coexist on the same individual [27]. This is because plant growth leads to gradual increase in the number of microhabitats for nesting sites [81], and in the abundance of available food resources: liquids secretions (nectaries and honeydew-producing hemipterans) [25], [26] and herbivore insects (important resource for arboreal predatory ants) [82].

Finally, there still remains a degree of uncertainty about the true role of species-area relationships in ecological ant-plant networks, and thus future studies should evaluate the effect of plant height on ant-plant networks in structurally more complex environments.

In summary, we demonstrate that (1) our individual-based ant-plant networks exhibited similar patterns of interactions when compared with ant-plant networks studied at the community level, suggesting that ant-plant interactions are much more dynamic than expected *a priori*, since ant species can change their role in network structure between diurnal and nocturnal period; (2) although plants with higher numbers of EFNs do not exhibit greater richness of foraging ants, our study produces basic information that increases our confidence in how individual-based ant-plant networks are structured, and the need to consider nocturnal records in ant-plant network sampling design so as to decrease inappropriate inferences; and (3) even though there is still much to do in order to have a better knowledge of the patterns and processes related to intrapopulation ant-plant networks, mainly to the true role of ant dominance hierarchy and availability of food resources in such networks, many things can be done when our suggestions are considered.

## Supporting Information

Appendix S1 **Species codes of ants recorded in two time intervals (D =  diurnal. N =  nocturnal) foraging on individuals of (C)** ***Chamaecrista mucronata*** **(Leguminosae–Caesalpinioideae), (S)** ***Stachytarpheta glabra*** **(Verbenaceae), and (Q)** ***Qualea grandiflora*** **(Vochysiaceae) in the Brazilian Neotropical Savanna.** Please see Figure 1 and text for more information.
(DOCX)
