# Supplementary material for: Individual-Based Ant-Plant Networks: Diurnal-Nocturnal Structure and Species-Area Relationship
Source: PLoS One. 2014 Jun 11;9(6):e99838. doi: 10.1371/journal.pone.0099838 (PMC4053393; doi:10.1371/journal.pone.0099838)
Supplement: Appendix S1 — Species codes of ants recorded in two time intervals (D = diurnal. N = nocturnal) foraging on individuals of (C) Chamaecrista mucronata (Leguminosae–Caesalpinioideae), (S) Stachytarpheta glabra (Verbenaceae), and (Q) Qualea grandiflora (Vochysiaceae) in the Brazilian Neotropical Savanna. Please see Figure 1 and text for more information. (DOCX) [file pone.0099838.s001.docx]

**SUPPORTING INFORMATION**

**Appendix S1**

Species codes of ants recorded in two time intervals (D= diurnal. N= nocturnal) foraging on individuals of (C) *Chamaecrista mucronata* (Leguminosae–Caesalpinioideae), (S) *Stachytarpheta glabra* (Verbenaceae), and (Q) *Qualea grandiflora* (Vochysiaceae) in the Brazilian Neotropical Savanna. Please see Figure 1 and text for more information.

| **Subfamily** | **Ant species** | **Species code** | **Time recorded** | **Plant associated** |
| --- | --- | --- | --- | --- |
| Formicinae | *Brachymyrmex* sp1 | B1 | D, N | Q |
| Formicinae | *Brachymyrmex heeri* | Bh | D | C |
| Formicinae | *Brachymyrmex pictus* | Bp | D, N | C, S |
| Formicinae | *Camponotus atriceps* | Ca | D, N | S |
| Formicinae | *Camponotus crassus* | Cc | D, N | C, S, Q |
| Formicinae | *Camponotus melanoticus* | Cm | D, N | C, S |
| Formicinae | *Camponotus novogranadensis* | Cn | D, N | C, S |
| Formicinae | *Camponotus renggeri* | Cr | N | S |
| Formicinae | *Camponotus rufipes* | Cf | N | C |
| Formicinae | *Camponotus senex* | Cs | D, N | C |
| Formicinae | *Camponotus* sp1 | CS1 | N | S |
| Myrmicinae | *Cephalotes atratus* | Ct | D, N | Q |
| Myrmicinae | *Cephalotes pusillus* | Cp | D, N | C |
| Myrmicinae | *Cephalotes* sp1 | C1 | N | Q |
| Myrmicinae | *Crematogaster* sp1 | R1 | N | Q |
| Ectatomminae | *Ectatomma tuberculatum* | Et | D, N | Q |
| Dolichoderinae | *Linepithema micans* | Lm | D, N | S |
| Formicinae | *Myrmelachista catarinae* | Mc | D, N | C, S |
| Formicinae | *Myrmelachista nodigera* | Mn | N | S |
| Paraponerinae | *Paraponera clavata* | Pc | N | Q |
| Myrmicinae | *Pheidole fallax* | Pf | D, N | S |
| Myrmicinae | *Pheidole* sp1 | P1 | D, N | Q |
| Myrmicinae | *Pheidole* sp2 | P2 | D, N | Q |
| Pseudomyrmecinae | *Pseudomyrmex gracillis* | Yg | D | S |
| Pseudomyrmecinae | *Pseudomyrmex pallidus* | Yp | N | C |
| Pseudomyrmecinae | *Pseudomyrmex* sp1 | Y1 | D | Q |
| Pseudomyrmecinae | *Pseudomyrmex* sp2 | Y2 | D, N | Q |
| Pseudomyrmecinae | *Pseudomyrmex* sp3 | Y3 | D, N | Q |
| Pseudomyrmecinae | *Pseudomyrmex* sp4 | Y4 | N | Q |
| Pseudomyrmecinae | *Pseudomyrmex termitarius* | Yt | D, N | C |
| Myrmicinae | *Solenopsis* sp1 | S1 | N | S |
| Myrmicinae | *Wasmannia auropunctata* | Wa | D, N | S |
